# Novel photodynamic therapy using two-dimensional NiPS_3_ nanosheets that target hypoxic microenvironments for precise cancer treatment

**DOI:** 10.1515/nanoph-2022-0520

**Published:** 2022-12-01

**Authors:** Zongze Wu, Quan Liu, Swelm Wageh, Zhe Sun, Omar A. Al-Hartomy, Abdullah G. Al-Sehemi, Lesen Yan, Jiaojuan Chen, Wenjian Zhang, Jilin Yang, Han Zhang, Liping Liu

**Affiliations:** Division of Hepatobiliary and Pancreas Surgery, Department of General Surgery, Shenzhen People’s Hospital (The Second Clinical Medical College, Jinan University; The First Affiliated Hospital, Southern University of Science and Technology), Shenzhen 518020, Guangdong, P. R. China; Key Laboratory of Optoelectronic Devices and Systems of Ministry of Education and Guangdong Province, College of Physics and Optoelectronic Engineering, and Otolaryngology Department and Biobank of the First Affiliated Hospital, Health Science Center, Shenzhen Second People’s Hospital, Shenzhen University, Shenzhen 518060, Guangdong, P. R. China; Department of Physics, Faculty of Science, King Abdulaziz University, Jeddah 21589, Saudi Arabia; Research Center for Advanced Materials Science (RCAMS), King Khalid University, P.O. Box 9004, Abha 61413, Saudi Arabia; Department of Chemistry, College of Science, King Khalid University, P.O. Box 9004, Abha 61413, Saudi Arabia

**Keywords:** mitochondria, photodynamic therapy, NiPS_3_, photosensitizer, ROS

## Abstract

Photodynamic therapy (PDT) is a highly promising modality against cancer, but its efficacy is severely limited by the low oxygen content in solid tumors. In this study, a smart photosensitive NiPS_3_ nanosheet was developed to solve the problem of low oxygen to allow PDT to be performed against tumors. The photosensitized ROS generation mechanism of NiPS_3_ is the photon-generated electron-hole pathway, which can generate O_2_^·−^ and ·OH at the conduction band and valance band, respectively. More crucial is that ·OH generation doesn’t need O_2_, and the O_2_^·−^ can also work in a low O_2_ environment, and depleting oxygen in tumor cells. Modified with triphenylphosphine (TPP) and based on density functional theory (DFT) calculations and experimental data, the NiPS_3_@TPP nano-system underwent targeted action toward mitochondria. *In vitro* experiments demonstrated that the reactive oxygen species (ROS) produced by NiPS_3_@TPP altered mitochondrial membrane permeability, which not only prolonged the PDT effect but also resulted in mitochondria apoptosis pathways inducing an apoptosis cascade. *In vivo* experiments demonstrated the targeting capability with low toxicity of the NiPS_3_@TPP nano-system. Tumor targeting at the tested dose indicated that it represented a promising biocompatible photosensitizer for *in vivo* biomedical applications.

## 1 Introduction

Photodynamic therapy (PDT) is a novel anti-cancer strategy in which photosensitizing agents selectively kill tumor cells by generating ROS when exposed to a suitable wavelength of light [1–3]. However, the therapeutic effects of PDT are significantly limited in a hypoxic tumor microenvironment since currently-available photosensitizers require oxygen to generate cytotoxic ROS [4, 5], including superoxide radicals (O_2_^·−^), singlet oxygen (^1^O_2_), hydrogen peroxide (H_2_O_2_) and hydroxyl radicals (·OH) [6]. Studies indicated that, even under severe hypoxic environment (2% O_2_), some photosensitive materials can generate considerable O_2_^·−^ through type I photoreactions, and partial O_2_^·−^ is transformed to high toxic ·OH through SOD-mediated cascade reactions. These radicals synergistically damage the intracellular organelles, which subsequently trigger cancer cell apoptosis, presenting a robust hypoxic PDT potency [7]. ·OH radical is one of the most damaging radical of all oxidants, having an oxidation potential (2.8 V) second only to that of fluorine, and relatively less dependent on molecular oxygen compared with other radicals [6, 8, 9]. Furthermore, it can trigger a rapid chain reaction with the majority of organic molecules in cells due to its high oxidation potential, oxidizing them to CO_2_ and H_2_O without the formation of secondary polluting reactants [10, 11]. Therefore, the efficacy of PDT can be substantially improved by generating large quantities of ·OH.

Various strategies have been utilized to alleviate treatment of disease [12–14]. Transition metal sulfides with narrow bandgap energy of approximately 0.2–2.0 eV, have been widely used in photocatalysis [15, 16]. One research found that NiPS_3_ was a more cost-effective platform for advancing photocatalytic [17]. Photocatalysis for water splitting is an effective strategy to realize O_2_ generation with H_2_O as a source [18, 19]. The flexible application of such a strategy for endogenous oxygen production precisely solves the problem of hypoxia in tumor microenvironments during the PDT of solid tumors [20]. Recent research has shown that a new nickel-related photosensitizer Ni_3_S_2_/Cu1.8S, which has a narrow bandgap, can stimulate the creation of electron-holes when combined with near infrared (NIR) irradiation [21]. For photosensitizers, the position of the conduction band minimum (CBM) and valence band maximum (VBM) relative to the hydrogen and oxygen electrode potentials is relevant [14, 22, 23]. Zhang et al. and Chen et al. have shown that NiPS_3_ nanomaterials are layered metal thiophosphites (MPS_3_), and can operate as semiconductor photosensitizers. NiPS_3_ can easily be prepared with high light stability compared with organic photosensitizers [24, 25]. NiPS_3_ has a puckered rather than planar structure, similar to that of phosphorene, two-dimensional (2D) NiPS_3_ monolayers that function indirectly, with an energy bandgap of 1.63 eV [26]. NiPS_3_ possesses [P_2_S_6_]^4−^ clusters that ‘ionically’ interact with Ni^2+^ within the material. Doping with NiPS_3_ electrons (0.1 e− per atom) which results in an increased (8.01 eV) van der Waals gap while doping the holes causes the gap to be reduced (3.20 eV). The addition of an electron elongates the Ni–S bond length, while the opposite effect has been observed when doping the holes. Very little changes are observed in P–S and P–P bond lengths during electron and hole doping [27]. In addition, NiPS_3_ nanomaterials have an adjustable bandgap that can generate O_2_^·−^ and ·OH when irradiated by laser light through the interaction of electron-hole pairs (e− – H+) and oxygen or water [22, 28], making it possible to perform PDT both in normoxic and hypoxic conditions. NiPS_3_ nanomaterials have gained considerable attention in PDT due to their biosafety, with a toxicity profile similar to that of black phosphorous, and significantly less toxic than CoPS_3_ or FePS_3_, indicating good biocompatibility [29–31]. Owing to their extremely short diffusion range (∼20 nm) and half-life (<200 ns), ROS is more effective when produced in critical sites such as the mitochondria or nucleus rather than the cytoplasm or other organelles [32, 33]. However, the majority of nanomaterials cannot pass through nuclear pores [34]. Mitochondria, on the other hand, are involved in multiple physiological and pathological processes [35], and therefore represent a viable therapeutic target. Furthermore, they are the cellular powerhouse that produces ATP to sustain cellular activities and survival, and also the primary site of ROS production. High levels of free radicals can trigger oxidative stress in the mitochondrial matrix, leading to the depolarization of mitochondrial membranes leading ultimately to apoptosis [36]. The mitochondrial electron transport chain is a major source of reactive oxygen species (ROS) and is also a target of ROS [37]. Meanwhile, the modification of positively charged triphenylphosphine (TPP) ligand on the surface of NiPS_3_ provided the mitochondrial-targeting property for the NiPS3@TPP by taking advantage of negative mitochondrial membrane potential [38, 39]. Consequently, the development of nano-systems that target organelles such as mitochondria during PDT is a promising therapeutic approach.

Thus, a safe and effective NiPS_3_-based PDT nano-system was developed, able to produce a large quantity of ROS in both normoxic and hypoxic conditions. In the present study, surface modification with TPP drove NiPS_3_ passage through the mitochondrial membrane via a delocalized positive charge, resulting in depolarization of the mitochondrial membrane and apoptosis of tumor cells [40]. More surprisingly, the cytotoxicity of NiPS_3_ can be revealed only when stimulated by specific wavelengths of light. If the light stimulus was discontinued, the cytotoxicity of NiPS_3_ also disappeared simultaneously. The strength of the NiPS_3_ toxicity was related to the power of the external light source, making it possible to regulate the NiPS_3_@TPP nano-system in clinical applications.

## 2 Materials and methods

NiPS_3_ nanosheets were provided by Shenzhen University, Guangdong, China. AO/PI was purchased from Logos Biosystems (South Korea), while the mitochondrial membrane potential assay kit (JC-1), Annexin V-FITC/PI apoptosis detection kits, and primary and secondary antibodies were obtained from Thermo Fisher (USA). Enhanced chemiluminescent (ECL) detection system kits were obtained from Tanon (Shanghai, China). Female Balb/c mice (15–20 g) were purchased from the Institute of Model Zoology, Nanjing University and were bred in a specific pathogen-free (SPF) environment.

### 2.1 Preparation of NiPS_3_ nanosheets

NiPS_3_ nanosheets were prepared by the synthesis of bulk NiPS_3_ crystals followed by electrochemical exfoliation, as described previously [22].

### 2.2 Characterization of NiPS_3_ nanosheets

X-ray diffraction (XRD) was performed using a Philips X’Pert Pro Super diffractometer with Cu Kα radiation. X-ray photoelectron spectroscopy (XPS) was conducted using an ESCALAB MK II with Mg Kα as the excitation source. Atomic force microscopy (AFM) was performed using a Veeco DI Nanoscope Multi Mode V system. UV–Vis DRS was performed using a Hitachi U-3010 UV–Vis spectrometer, with absorption spectra recorded on a PerkinElmer Lambda 950 UV−vis−NIR spectrophotometer [22].

### 2.3 Mechanisms for ·OH production ESR experiment

The ESR experiment was primarily focused on the analysis of ·OH. In the ·OH experiment, 5 mg of NiPS_3_ were dispersed in 5 mL water via ultrasound for 20 min. Argon was then bubbled through the solution for 20 min to remove oxygen. DMPO (100 mM) solution (also bubbled with argon to remove oxygen) was used for hybrid acquisition within 660 nm light. Simultaneously, the same experiment was performed in normoxic conditions as a control. All tests were conducted in weak acidic conditions (pH = 6.8). Finally, whether H+ was able to generate ·OH was determined [41].

### 2.4 Preparation of the NiPS_3_@TPP system

A 1:1 mass ratio of NiPS_3_ to TPP was dissolved in anhydrous ethanol and fully dissolved while exposed to ultrasound for 30 min to form the NiPS_3_@TPP targeting nano-system. The zeta potentials of NiPS_3_ and NiPS_3_@TPP were measured using a Malvern Mastersizer 2000 (Zetasizer Nano ZS90, Malvern Instruments Ltd, UK).

### 2.5 Cell culture

Huh-7 human liver cancer cells and LO_2_ were obtained from the Shanghai Cell Bank, Chinese Academy of Sciences, and cultured in Dulbecco’s modified Eagle’s medium (DMEM) supplemented with 10% fetal bovine serum (FBS) and 1% antibiotics (penicillin-streptomycin, 10,000 U/mL) at 37 °C within an atmosphere containing 5% CO_2_. Normoxic and hypoxic conditions were simulated by culturing the cells within 21 and 1% O_2_, respectively. Hypoxia preconditioning performed using tri-gas incubator (Smartor118, Ningbo Huayi Ningchuang Intelligent Technology Co., LTD).

### 2.6 *In vitro* toxicity of NiPS_3_ nanosheets

Huh-7 and LO_2_ cells were seeded at a density of 8000 cells per well in 96-well plates and incubated overnight. The cells were then incubated with NiPS_3_ and NiPS_3_@TPP at different concentrations (0, 12.5, 25, 50, 100, and 200 ppm) for 12 and 24 h (*n* = 5). The culture medium was replaced with CCK-8 reagent, and the cells were incubated for an additional 1 h at a predetermined time. The absorbance at 450 nm was measured using a microplate reader (ELx808; BioTek). Untreated cells were used as control. Normoxic and hypoxic conditions were simulated by culturing the cells within 21 and 1% O_2_, respectively.

To explore the optimal power for PDT, the 660 nm laser power was set to 0, 0.1, 0.2, 0.3, 0.4, and 0.5 W/cm^2^. The viability at each setting was determined using a CCK-8 assay. Finally, the NiPS_3_@TPP photodynamic activity (1% O_2_) was tested at 12, 24, 48, and 72 h.

### 2.7 AO/PI staining

Huh-7 cells were seeded into 96-well plates and cultured overnight, then incubated with 100 ppm NiPS_3_ or NiPS_3_@TPP for 6 h. After irradiation with a 660 nm laser at 0.3 W/cm for 10 min, the cells were stained with acridine orange (AO, green, live cells) and propidium iodide (PI, red, dead cells) purchased from Logos Biosystems (South Korea), as per standard protocols. The cells were rinsed three times with PBS and observed using a confocal microscope to assess cell viability.

### 2.8 *In vitro* photodynamic effects

Huh-7 cells were seeded into 96-well plates, cultured overnight, and then cultivated with NiPS_3_ for 6 h. The oxygen content within the culture environment was adjusted in the incubator, normoxic and hypoxic conditions represented by 21% O_2_ and 1% O_2_, respectively. 2,7-Dichlorodihydro-fluorescein·diacetate (DCFH-DA) probe dissolved in DMEM without FBS at a concentration of 1:1500 was added to the plates, then incubated for 30 min prior to irradiation with a 660 nm laser at 0.3 W/cm^2^ for 10 min. The cells were rinsed three times with PBS then observed using a confocal microscope.

### 2.9 Scanning electron microscopy

Huh-7 cells were seeded in 6-well plates and cultured until 80% confluent. The culture medium was replaced with 2 mL fresh DMEM medium supplemented with 100 ppm NiPS_3_. Three groups: (A) NiPS_3_, (B) NiPS_3_@TPP, and (C) NiPS_3_@TPP + 660 nm, were tested, respectively. After incubation for 6 h, the experimental groups were irradiated with a 660 nm laser at 0.3 W/cm^2^ for 10 min and cultured for a further 12 h. The cells were then harvested, fixed, and sectioned prior to observation using a scanning electron microscope.

### 2.10 Mitochondrial membrane potential (△Ψm) measurements

Huh-7 cells were seeded in 24-well plates at a density of 5 × 10^5^ cells/well and cultured until 80% confluent. After incubation with the NiPS_3_@TPP nano-system for 6 h, then laser irradiated at 0.3 W/cm^2^ for 10 min, the cells were washed with PBS then stained using a mitochondrial membrane potential assay kit (JC-1) (Thermo Fisher, M34152) in accordance with the manufacturer’s instructions. The stained cells were analyzed by flow cytometry (BD FACSAria).

### 2.11 Annexin staining

Huh-7 cells were seeded in 24-well plates at a density of 5 × 10^5^ cells/well and cultured until 80% confluent. After incubation with the NiPS_3_@TPP nano-system for 6 h, then laser irradiated at 0.3 W/cm^2^ for 10 min, the cells were washed with PBS prior to staining using an Annexin V-FITC kit (Thermo Fisher,V13242), in accordance with the manufacturer’s instructions. Early and late apoptotic cells were analyzed by flow cytometry (BD FACSAria).

### 2.12 Western blot analysis

Total proteins were extracted from suitably treated cells using RIPA lysis buffer (Thermo Fisher, 89,900), the concentration of which was determined using a bicinchoninic acid (BCA, Thermo Fisher, 23,227) protein assay kit. Cytoplasmic cytochrome C was extracted using a mitochondria isolation kit (Thermo Fisher, 89,874), in accordance with the manufacturer’s instructions. Antibodies against cytochrome C (Thermo Fisher, 33–8500), Bcl-2 (Thermo Fisher, MA5-41210), and caspase-3 (Thermo Fisher, MA1-91637) were used to measure protein expression, with β-actin (Thermo Fisher, MA5-15452) as the internal reference, as described previously by Liu [42].

### 2.13 γ-H_2_AX foci assay

Huh-7 cells were cultured on coverslips in six-well plates to 80% confluency, and then incubated with NiPS_3_ or NiPS_3_@TPP for 4 h prior to irradiation with 660 nm laser light for 10 min at a light density of 0.3 W/cm^2^. Four hours after irradiation, the cells were fixed with 4% paraformaldehyde for 10 min. The cells were then permeabilized with 0.2% Triton X-100 for 15 min, incubated in blocking buffer (3% bovine serum albumin in PBS) for 45 min, then washed with PBS and incubated with a primary monoclonal antibody against γ-H_2_AX (Abcam, ab81299) at 4 C overnight. The cells were then washed with PBS and incubated with a FITC-labeled goat anti-rabbit IgG secondary antibody (Abcam, ab97050) for 1 h. The nuclei were additionally stained with DAPI for 10 min. γ-H_2_AX foci were imaged via confocal laser-scanning microscopy (CLSM).

### 2.14 *In vitro* bio-distribution

To detect the *in vitro* Bio-distribution, NiPS_3_ and NiPS_3_@TPP were labeled with PEG-Cy7 and incubated with Huh-7 cells in a 27 mm confocal petri dish. After that, Hoechst 33,342 (beyotime.com, C1027) and Mito-Tracker Green (beyotime.com, C1048) were added for staining cell nucleus and mitochondria, respectively. The confocal microscopy (CLSM, Zeiss 710 NLO) was used to take fluorescent photographs of the stained cells.

### 2.15 *In vivo* biodistribution

For the *in vivo* fluorescence imaging experiments, the BALB/c (nu/nu) mice with Huh-7 tumors were divided into two groups (*n* = 3 in each group). NiPS_3_ and NiPS_3_@TPP were labeled with PEG-Cy7. The mice were then injected via their tails vein with NiPS_3_/Cy7 and NiPS_3_@TPP/Cy7 (dose: 100 μL, 5 mg/kg bodyweight), respectively. An IVIS Spectrum (PerkinElmer) was used to obtain *in vivo* images of the mice at 12 and 24 h postinjection. A 740 nm wavelength light was used as the excitation source, and 760 nm emitted light was detected. Afterward, mice were sacrificed, and the tumor, heart, liver, spleen, lung, and kidney were collected. The fluorescence in all organs was acquired using the IVIS Spectrum (PerkinElmer) imaging system.

### 2.16 *In vivo* toxicity evaluation

Female Balb/c mice (15–20 g) were purchased from the Institute of Model Zoology, Nanjing University. All experiments were performed in accordance with the guidelines of the National Regulations of China for the Care and Use of Laboratory Animals. The mice were injected with 3 mg/kg NiPS_3_ or isotonic saline (100 μL), NiPS_3_ was dissolved in saline and injected via tail vein. The duration of the toxicity test was 1, 7, or 14 days, respectively. On the last day, 0.8 mL of blood was sampled from the eyes of the mice. A blood routine test (BRT) was conducted at the Shenzhen Sun Yat-sen Cardiovascular Hospital. The heart, liver, spleen, lungs, and kidneys of the mice were resected and stained with hematoxylin and eosin (H & E) on the fourteenth day.

### 2.17 Establishing a xenograft liver tumor model and *in vivo* PDT

Huh-7 cells (1.0 × 10^6^ cells, 100 μL) were injected into the right armpit of each mouse (as described for the *in vivo toxicity evaluation* experiment), and after the tumors had grown to 4–6 mm in diameter, the mice were randomly divided into one of 6 groups: (1) Control (saline); (2) 660 nm laser illumination; (3) NiPS_3_; (4) NiPS_3_ + 660 nm laser; (5) NiPS_3_@TPP; (6) NiPS_3_@TPP + 660 nm laser (*n* = 5 per group). The dosage of NiPS_3_@TPP particles was 3 mg/kg and sample was dissolved in saline and injected via tail vein. Ten hours after injection, the tumors were irradiated for 10 min with a 660 nm laser at 0.3 W/cm^2^. PDT was performed 3 times with gaps of 2 days between treatments. The volumes of tumors and body weights of the mice were recorded every 2 days for 14 days. The perpendicular diameters of the tumors were measured using calipers, and the volume (mm^3^) was calculated as 0.5 × length × width^2^.

### 2.18 Statistical analysis

Data were presented as means ± standard deviation of three independent experiments. All statistical analyses were conducted using OriginPro-8 software. *P*-values < 0.05 were considered statistically significant.

## 3 Results

### 3.1 Morphological and physical property characterization

**Scheme 1:**
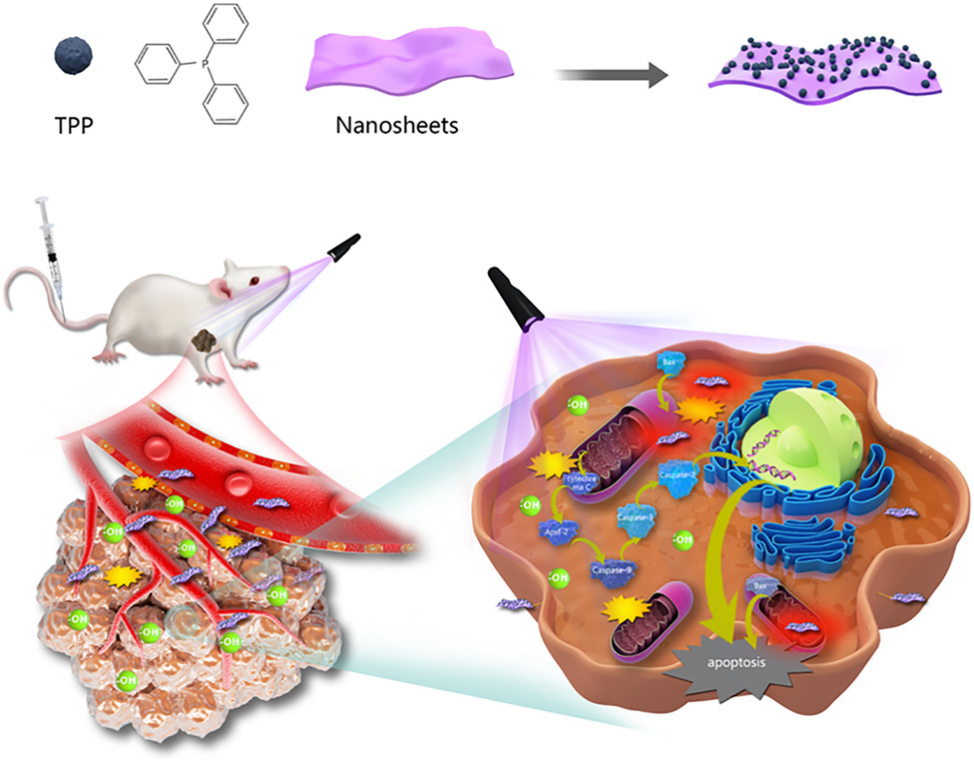
Graphic illustration describing the structure of NiPS_3_@TPP and its roles in hypoxic photodynamic therapy.

**Figure 1:**
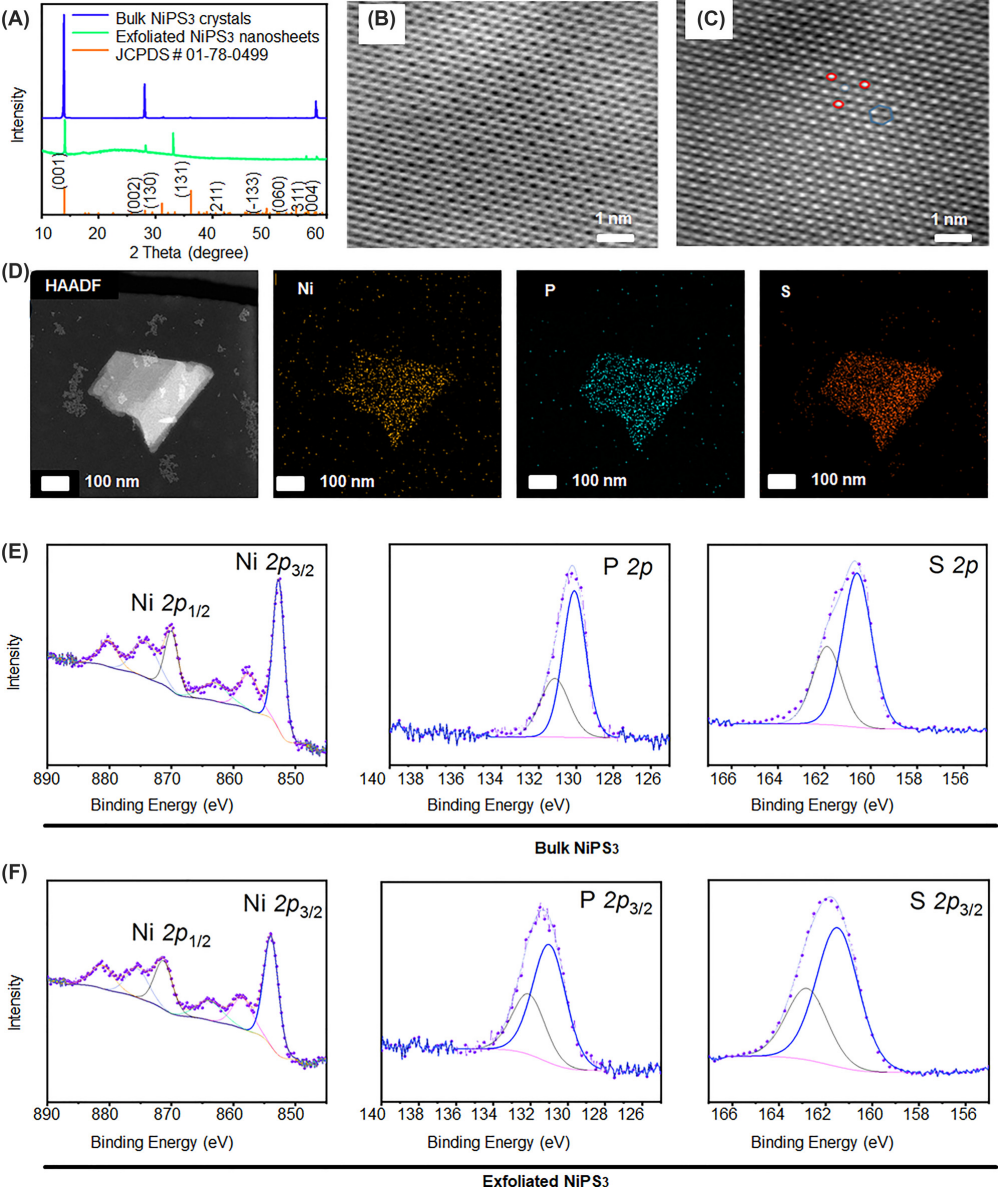
Characterization of NiPS_3_. (A) X-ray diffraction pattern of NiPS_3_ samples. (B–C) STEM-BF and STEM-HAADF images of NiPS_3_. (D) TEM-EELS mapping of exfoliated LSTL NiPS_3_. (E) XPS spectra of bulk NiPS_3_. (F) XPS spectra of exfoliated NiPS_3_.

In the present study, NiPS_3_@TPP produced ROS in mitochondria, which induced apoptosis, as displayed in the schematic diagram (Scheme 1). As shown in Figure S1, the NiPS_3_ particles crystallized into space group *C*2/*m* (No. 12) with triclinic unit cell dimensions of *a* = 5.812 Å, *b* = 10.070 Å, *c* = 6.632 Å, and *V* = 371.2 Å^3^. This is an ideal structure for splitting water due to fully exposed S and P atoms that form an octahedron [22, 28, 43]. These parameters were shown by Li [22] and summarized in Supplementary Table 1. In this model, the Ni ions were immobilized on a [P_2_S_6_]^4^- framework, with a metal layer sandwiched by distorted octahedral S layers forming a 2D structure stacked together via van der Waals forces [22]. These structural characteristics have important implications for its optical applications, which have been described in the journal, Nature *etc*. [27, 44]. As shown in Figure 1A, the X-ray diffraction (XRD) peaks of both bulk NiPS_3_ and ultrathin NiPS_3_ nanosheet correspond to the standard XRD spectrum (JCPDS# 01-78-0499), with no additional peaks observed. This indicated that pure phase NiPS_3_ was successfully synthesized and no impurities were introduced during electrochemical exfoliation. The ultrastructure of the NiPS_3_ nanosheets was analyzed using scanning transmission electron microscopy-bright field (STEM-BF) and scanning transmission electron microscopy-high-angle annular dark field (STEM-HAADF). The entire crystal domain had a homogeneous and almost flawless structure (Figure 1B and C). Furthermore, mapping by electron energy-loss spectroscopy in a transmission electron microscope (TEM-EELS) revealed a uniform distribution of Ni, P, and S elements over the entire exfoliated flake (Figure 1D). The XPS spectra of the bulk crystals and nanosheets revealed peaks of Ni2p (∼885–850 eV), P2p (∼130.9 eV), and S2p (∼161.3 eV). Gaussian fitting indicated that the ultrathin NiPS_3_ nanosheets displayed almost the same spectra as bulk NiPS_3_. Furthermore, peaks corresponding to oxidized P and S were not observed in the NiPS_3_ nanosheets, suggesting that negligible oxidation had occurred (Figure 1E and F).

**Figure 2:**
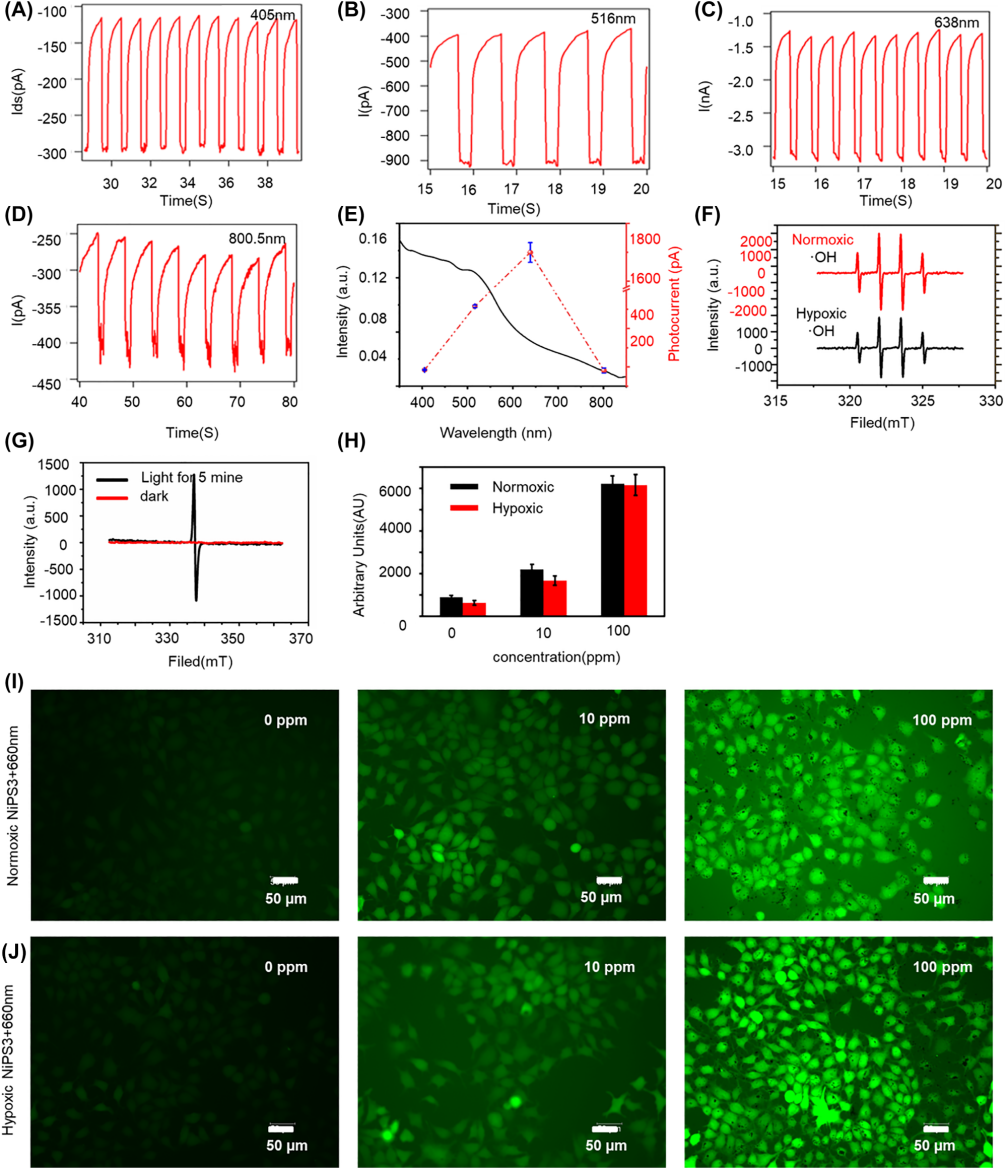
Production of ROS and optical properties of NiPS_3_. **(**A–D) Photoresponse of NiPS_3_ at different light wavelengths (405, 516, 638, and 800.5 nm). (E) UV–Vis DRS spectra of NiPS_3_. (F) ESR spectra of ·OH. (G) ESR spectra of NiPS_3_ recorded in the dark and illuminated for 5 min. (H) Fluorescence intensity of Figure 2I and J. (I–J) ROS levels represented by fluorescence intensity of DCFH-DA in Huh-7 cells incubated with NiPS_3_ and irradiation with 660 nm laser light (0.3 W/cm^2^, 10 min).

The optical absorption properties of a photosensitizer represent the key determinants of its photodynamic capabilities [45]. The photoelectric properties of NiPS_3_ make it possible to utilize in optically controlled applications. The photo-response of NiPS_3_ using 405, 516, 638, and 800.5 nm laser light are displayed in Figure 2A–D. It should be noted that the photo-response at 638 nm was beyond the individual response range, which suggested that this may be a key characteristic of NiPS_3_ regarding light control. The NiPS_3_ nanosheets absorbed light in the visible and near-infrared range (NIR), taking to account penetration during light therapy and exhibits absorption at 660 nm, NiPS_3_ nanosheets is suitable for photocatalytic activity in PDT (Figure 2E). To confirm the exact form of ROS, an ESR test was conducted. ESR spectroscopy further revealed the presence of DMPO-·OH under both normoxic or hypoxic conditions (Figure 2F), indicating that irrespective of whether it was utilized in normoxic or hypoxic conditions, NiPS_3_ in PDT was able to generate ·OH radicals. During hypoxia, with H+ generated by the NiPS_3_ nano-particles oxidizing OH− in water to produce ·OH, resulting in effective PDT even at low oxygen levels. The ESR spectrum of NiPS_3_ displayed distinct electron-hole pairs, indicative of the presence of ·OH. As shown in Figure 2G, the signal intensity of light was evidently strong in comparison with the peak for the same sample in darkness, indicating the presence of H+. Intracellular ROS generation was further evaluated using Huh7 cells as typical cancer cells (Figure 2I and J), Statistical analysis of fluorescence intensity data is shown in Figure 2H. Compared with the normoxic group, the hypoxic group also revealed DCFH-DA green fluorescence, demonstrating intracellular ROS was also producted in hypoxic condition. Compared with other 2D materials, NiPS_3_ is more stable, less toxic, and has greater catalytic activity (Supplementary Table 2).

### 3.2 *In vitro* cytotoxicity

**Figure 3:**
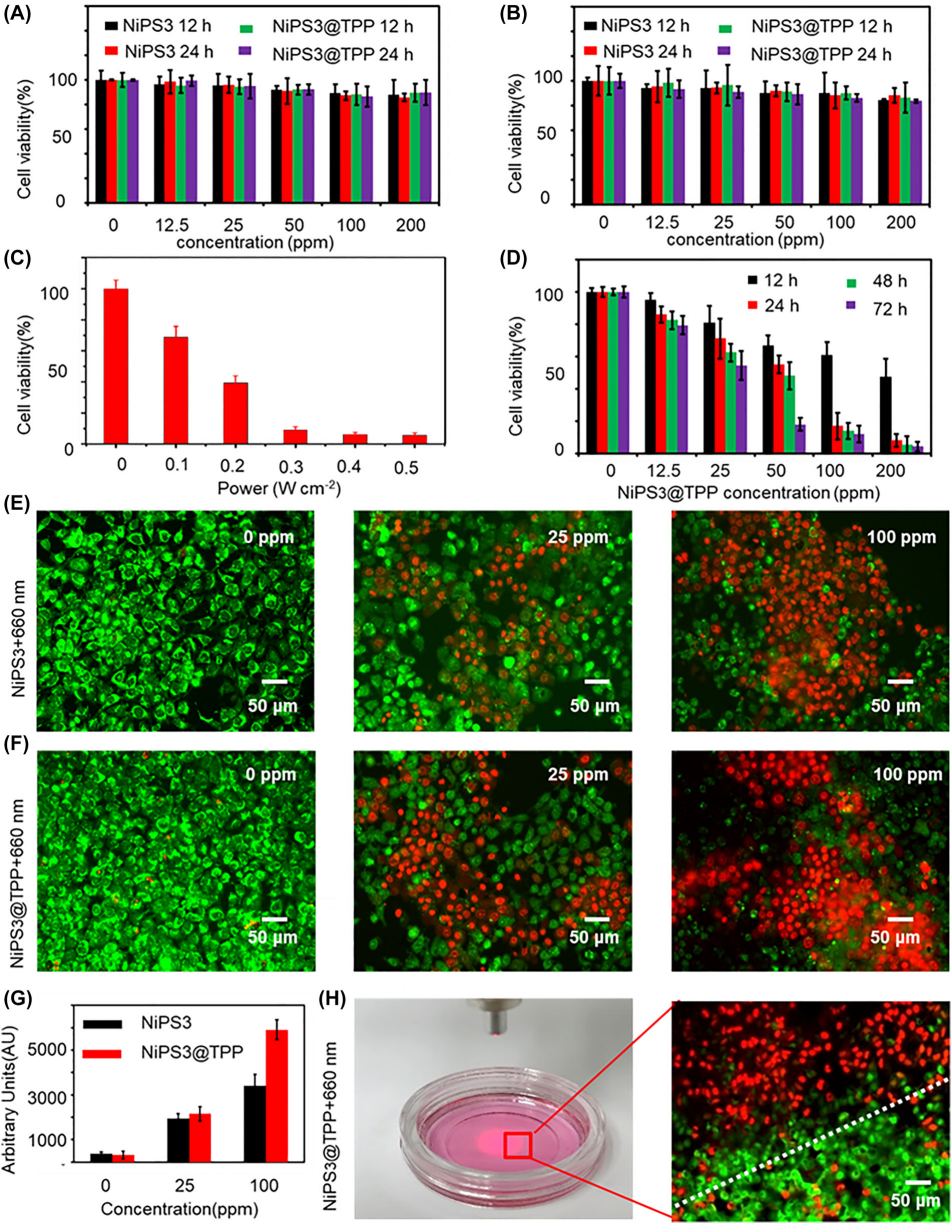
Biocompatibility and photodynamic effect of NiPS_3_. (A–B) Percentage of viable Huh-7 and LO_2_ cells after incubation with different concentrations of NiPS_3_ (0, 12.5, 50, 100, and 200 ppm) for 12 and 24 h, respectively. (C) Optimum photodynamic power. (D) Percentage of viable Huh-7 cells after incubation with different concentrations of NiPS_3_@TPP (0, 12.5, 50, 100, and 200 ppm) and photodynamic therapy with 660 nm laser light (0.3 W/cm^2^, 10 min, 1% O2). (E–F) Representative images of AO/PI stained cells; red and green indicate dead and living cells, respectively. (G) Fluorescence intensity quantified for dead cells of E and F. (H) Cell death caused by photodynamic therapy.

The biocompatibility and photodynamic effects of NiPS_3_ were then evaluated under hypoxic conditions (1% O_2_) to determine its feasibility as a photosensitizer for anti-tumor PDT. As shown in Figure 3A and B, in the absence of 660 nm laser irradiation, the viability of Huh-7 and LO_2_ cells was not significantly affected by 0–200 ppm NiPS_3_, even when incubated with NiPS_3_ for 24 h, indicating minimal cytotoxicity of the NiPS_3_ nanosheet, a result consistent with previous research [29, 46]. As shown in Figure 3C, its optimal power was achieved by irradiating at 0.3 W/cm^2^ for 10 min, a setting used for subsequent experiments. As shown in Figure 3D, NiPS_3_@TPP displayed a potent photodynamic effect against Huh-7 cells when irradiated with a 660 nm laser in a time and concentration-dependent manner. To directly observe cell death, acridine orange (AO, green) and propidium iodide (PI, red) were used to label living and dead cells. As displayed in Figure 3E and F, with increasing concentrations of NiPS_3_@TPP, green fluorescence was attenuated and red fluorescence increased in intensity, indicating cell death due to the generation of ROS. Image J was used to analyze the fluorescence (Red) intensity of dead cells, and the data analysis was shown in Figure 3G. To exclude the possibility of any inherent cytotoxicity of NiPS_3_@TPP, a proportion of the culture dishes were irradiated with 660 nm laser light only for 10 min, and the proportion of live and dead cells measured after staining with AO/PI. Only a few dead cells were observed in the non-photodynamic region, whereas almost all cells had died in the PDT region, as shown in Figure 3H. Similar results were obtained using electron microscopy, as shown in Figure S2. Under an inverted microscope, the effects of different concentrations of NiPS_3_@TPP on cell growth were observed. The results indicated that NiPS_3_ nanosheets exhibited low toxicity toward Huh7 cells. Taken together, the results suggested that NiPS_3_ nanosheets were biocompatible and effective at killing cells using PDT.

### 3.3 Tumor-targeting efficacy

**Figure 4:**
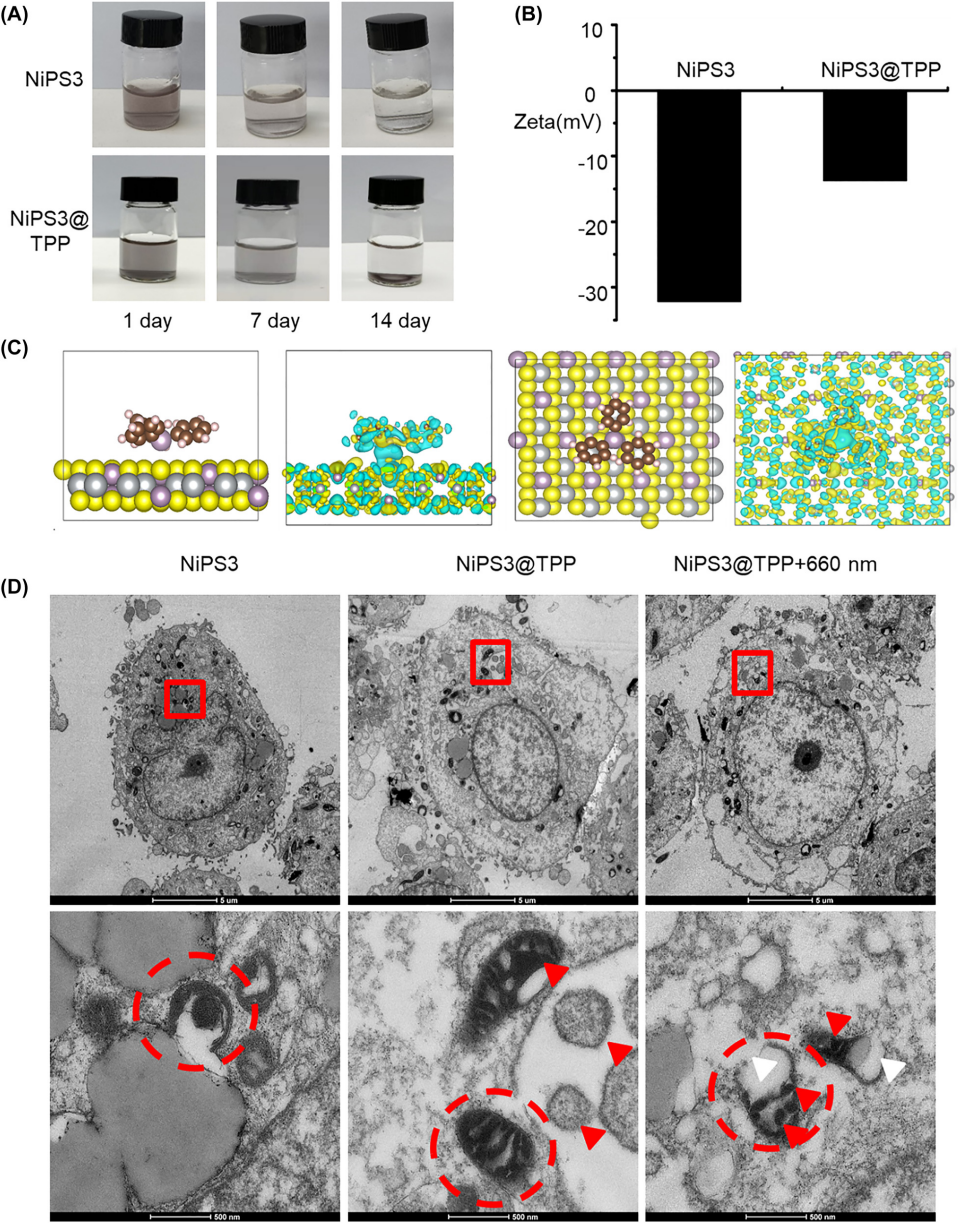
Properties and tumor targeting of NiPS_3_@TPP. (A) Dispersity of NiPS_3_ and NiPS_3_@TPP in water. (B) Zeta potential of NiPS_3_ and NiPS_3_@TPP. (C) Lattice and electron cloud side view of NiPS_3_@TPP. (D) Scanning electron microscopy research materials intracellular distribution in Huh-7 cells.

Due to the extremely short diffusion range and half-life of ROS, greater damage is caused if it is produced within key organelles such as mitochondria rather than in the cell membrane or cytoplasm. Mitochondria are the optimum site for PDT since the decrease in mitochondrial membrane potential (MMP) following excessive ROS production is irreversible, triggering apoptosis that can enhance the effect of PDT. Furthermore, as tumor cells require high quantities of energy for proliferation and drug resistance, targeting mitochondria can significantly impair their bioenergetic status [47]. Finally, mitochondrial targeting can also induce endogenous apoptosis by promotion of the release of cytochrome C from the depolarized membrane, therefore sensitizing the cells to PDT. Selective mitochondrial uptake of a drug can be achieved *in vivo* by linking a lipophilic cation to the molecule [48]. Therefore, TPP was attached to the surface of NiPS_3_ to target the mitochondria of tumor cells. This enhanced the efficiency of ROS in PDT. The stability of NiPS_3_ and NiPS_3_@TPP is shown in Figure 4A, their zeta potentials in Figure 4B, and the absorption electron cloud diagrams of TPP and NiPS_3_NiPS_3_ in Figure 4C.

In the present study, NiPS_3_@TPP produced ROS and induced apoptosis. Therefore, we explored the intracellular localization and photocatalytic activity of the NiPS_3_@TPP targeting system under hypoxic conditions. As shown in Figure 4D, Huh7 cells were incubated with NiPS_3_, NiPS_3_@TPP, and NiPS_3_@TPP + 660 nm. In NiPS_3_ group, the nanomaterials were mainly swallowed by lysosomes and randomly dispersed in cytoplasm. In the NiPS_3_@TPP group and the NiPS_3_@TPP + 660 nm group, nanomaterials showed obvious enrichment in mitochondria, and the nanomaterials were distributed in or around the inner mitochondrial membrane. However, when Huh7 cells were incubated with NiPS3@TPP + 660 nm, their mitochondrial inner membrane collapsed and shrank, with large vacuoles formed in the inner cavity and the outer membrane ruptured.

**Figure 5:**
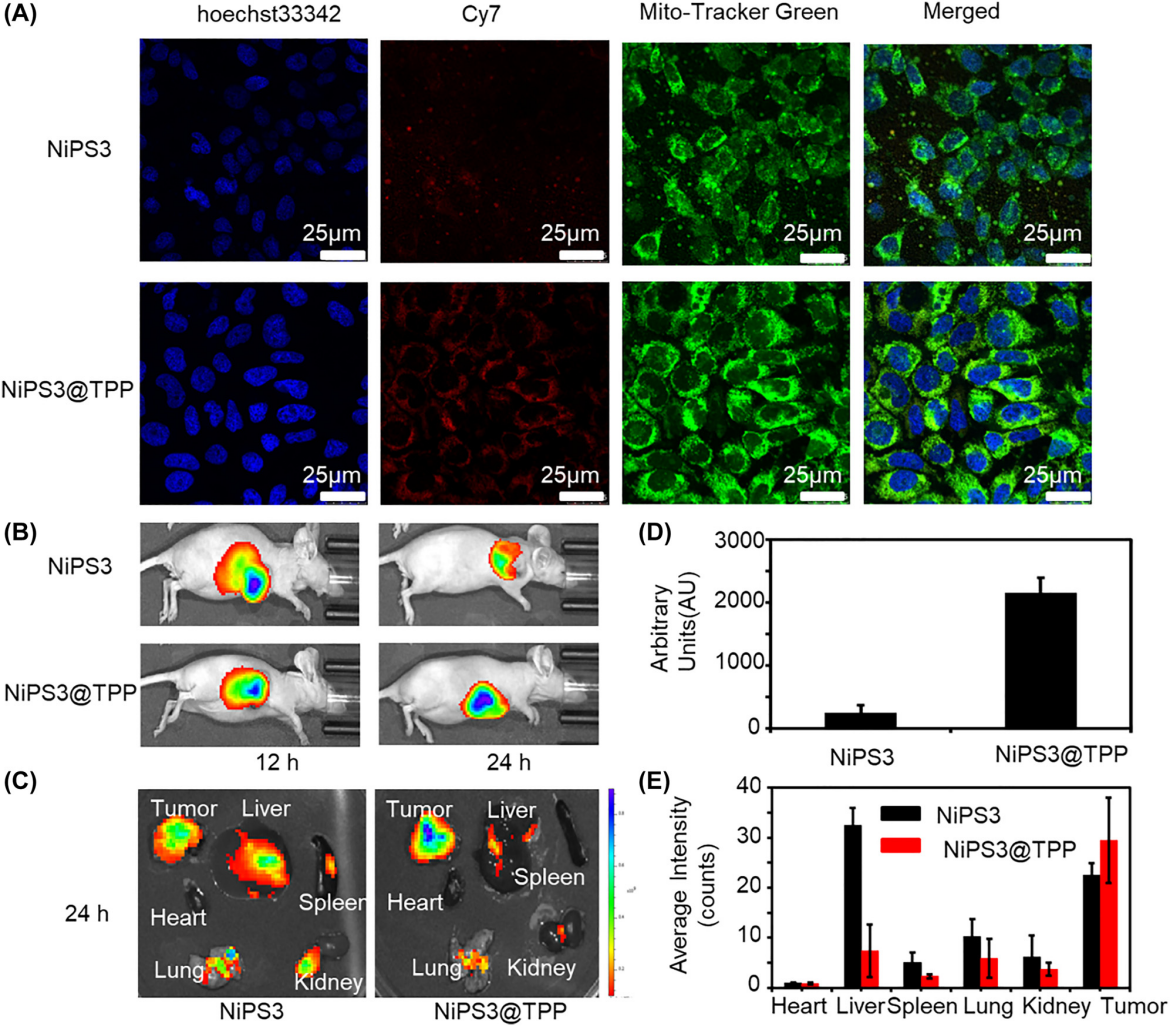
Biodistribution and *in vivo* imaging. (A) Confocal images of Huh-7 cell internalization of NiPS_3_@TPP/Cy7 (blue: cell nucleus stained with hoechst33342; green: mitochondria stained with Mito-Tracker green; red: NiPS_3_@TPP/Cy7), scale bar: 25 μm. (B) Semiquantitative biodistribution of NiPS_3_/Cy7 or NiPS_3_@TPP/Cy7 in BALB/C mice. (C) Fluorescence intensity of tumors and major organs. (D) Fluorescence intensity quantified for Cy7 *in vitro* targeting. (E) Fluorescence intensity quantified for Cy7 tumors and major organs.

Intracellular trafficking of NiPS_3_@TPP in Huh-7 cells was investigated by CLSM. Cy7-labeled NiPS_3_@TPP was efficiently internalized and localized in mitochondria compartments after incubation for 12 h, as demonstrated by colocalization of Cy7 with mitochondria label fluorescence, shown as yellow fluorescence in the merge image (Figure 5A). Compared with NiPS_3_, the mitochondria targeting of NiPS_3_@TPP were significantly increased, Fluorescence intensity quantified for Cy7 *in vitro* targeting were shown as (Figure 5D).

A Huh-7 xenograft tumor model was established for the investigation of *in vivo* therapeutic effects of NiPS_3_@TPP to explore its potential for further pre-clinical application. The *in vivo* biodistribution and tumor accumulation of NiPS_3_@TPP/Cy7 after intravenous injection were determined via *in vivo* fluorescence imaging. As shown in Figure 5B, in NiPS_3_/Cy7 group, fluorescent signals reduced significantly after 24 h, this was probably caused for the reason that the tumor was rich in blood supply and had relatively high blood drug concentration effect for a short time, but due to the lack of targeting, it was soon carried to other parts by the blood flow. The NiPS_3_@TPP/Cy7 group exhibited relatively high and stable accumulation levels in tumors at both measured time-points, which could generally be attributed to the enhanced permeability and retention effect (EPR). After reaching the blood vessels around the tumor, due to the mitochondrial targeting effect of TPP, the material could quickly pass through the blood vessels and the tumor cells, and then riveted in the tumor cells. In addition to fluorescent signals detected at tumor sites, they were also detected in the major mouse organs including the liver, kidney, and lung, shown as Figure 5C. Semiquantitative biodistribution of NiPS_3_/Cy7 and NiPS_3_@TPP/Cy7 in BALB/C mice detected by the average fluorescence intensity of tumors and major organs were shown as Figure 5E.

### 3.4 Mitochondrial targeting mechanism research

**Figure 6:**
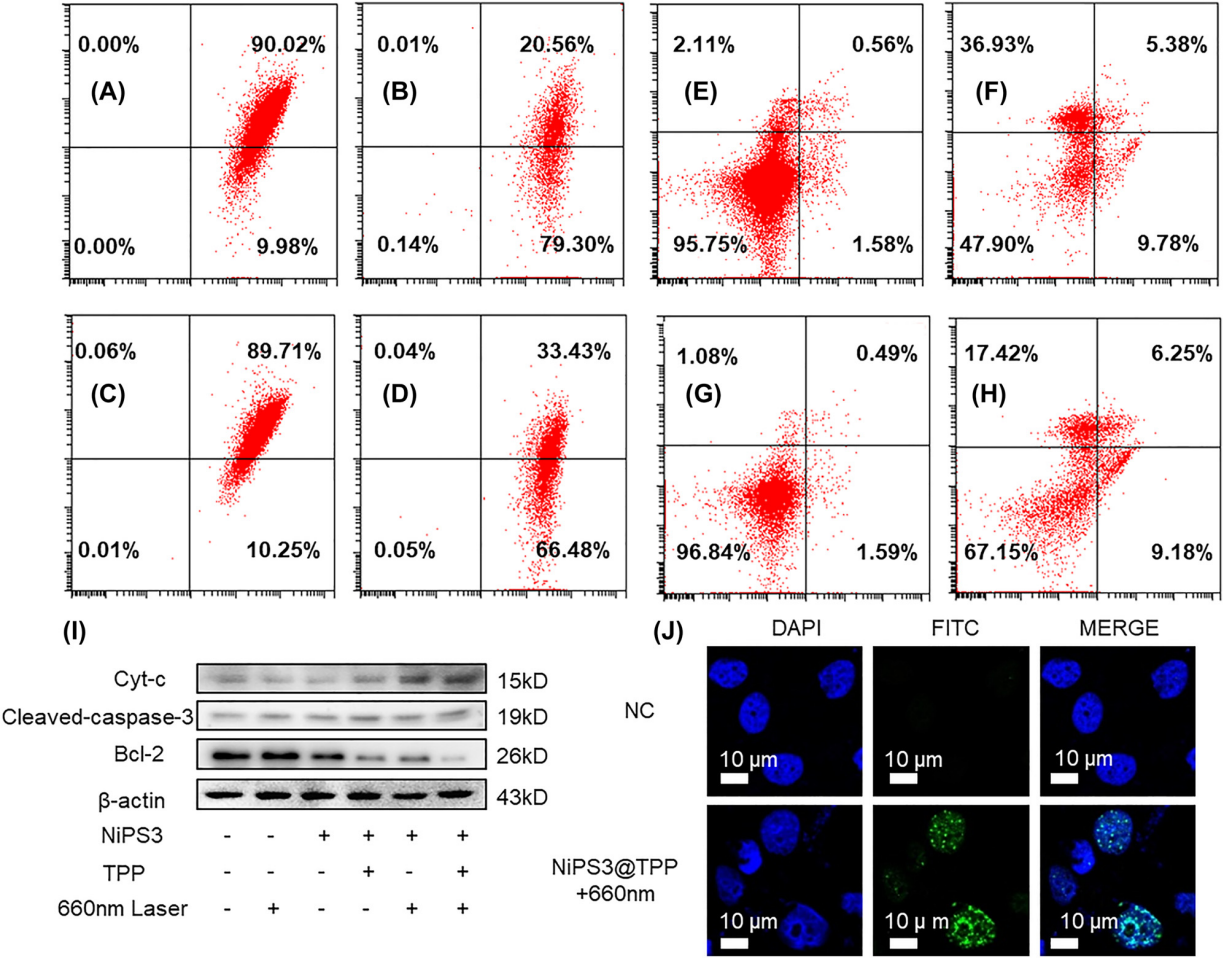
Photodynamic mechanism of NiPS_3_@TPP. (A) MMP measurement in normoxic conditions (21% O_2_), cultivation with NiPS_3_@TPP. (B) MMP measurement in normoxic conditions (21% O_2_), cultivation with NiPS_3_@TPP and PDT. (C) MMP measurement in hypoxic conditions (1% O_2_), cultivation with NiPS_3_@TPP. (D) MMP measurement in hypoxic conditions (1% O_2_), cultivation with NiPS_3_@TPP and PDT. (E) Measurement of apoptosis in normoxic condition (21% O_2_), cultivation with NiPS_3_@TPP. (F) Measurement of apoptosis in normoxic conditions (21% O_2_), cultivation with NiPS_3_@TPP and PDT. (G) Measurement of apoptosis in hypoxic conditions (1% O_2_), cultivation with NiPS_3_@TPP. (H) Measurement of apoptosis in hypoxic conditions (1% O_2_), cultivation with NiPS_3_@TPP and PDT. (I) WB showing expression levels of cytochrome C, cleaved-caspase-3, and Bcl-2 proteins in Huh-7 cells treated with 100 ppm NiPS_3_@TPP and irradiated with 660 nm laser light (0.3 W/cm^2^) for 10 min. (J) Representative immunofluorescence images showing γ-H_2_AX foci in Huh-7 cells.

Previous research have found that loss of mitochondrial membrane potential (MMP), mitochondrial depolarization, and cytochrome c (Cyt c) release induced cell apoptosis [49, 50]. Therefore, we investigated the PDT effect of NiPS_3_@TPP by measuring MMP and the rate of apoptosis. In normoxic conditions (21% O_2_), experimental group therapy at 660 nm, 0.3 W/cm^2^ for 10 min, the MMP of the experimental group (Figure 6B) decreased 69.32% compared with the control group (Figure 6A). In hypoxic conditions (1% O_2_), the MMP of the experimental group (Figure 6D) decreased 56.23% compared with the control group (Figure 6C). Although the decrease in MMP within hypoxic conditions was less than that observed in normoxia, the decrease was nevertheless significant. Similar results were also observed for the experiments of apoptosis. In normoxia, the proportion of dead and early/late-stage apoptotic cells in the experimental group (Figure 6F) was 52.09% higher than the control group (Figure 6E). In hypoxia (1% O_2_), the proportion of dead and early/late-stage apoptotic cells in the experimental group (Figure 6H) was 32.85% higher than that of the control group (Figure 6G). A significant increase in the expression levels of cytochrome C and pro-apoptotic cleaved-caspase 3, with a concomitant decrease in pro-survival Bcl-2 (Figure 6I) was observed. Furthermore, γ-H_2_AX was detected in the PDT group, indicative of DNA double-strand breaks caused by ROS (Figure 6J). Taken together, the results indicated that the NiPS_3_@TPP nano-system was capable of catalyzing the generation of ROS within both normoxic and hypoxic conditions.

### 3.5 *In vivo* anticancer experiments

**Figure 7:**
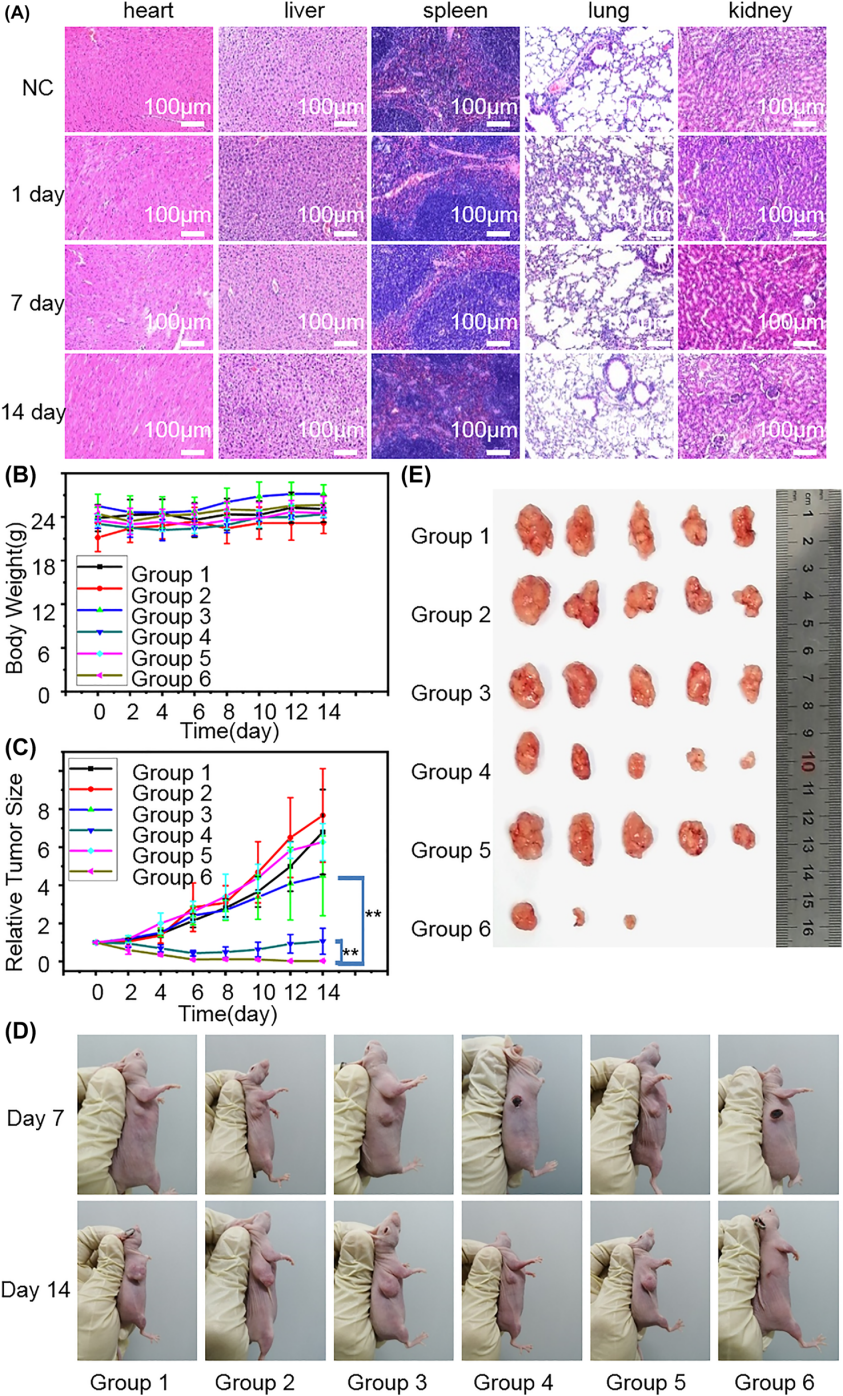
*In vivo* therapeutic effect of the NiPS_3_@TPP nanosystem combined with photodynamic therapy. Group 1: normal saline (control), group 2: 660 nm laser illumination, group 3: NiPS_3_, group 4: NiPS_3_ + 660 nm laser, group 5: NiPS_3_@TPP, group 6: NiPS_3_@TPP + 660 nm laser, PDT duration 10 min. (A) Intravenous injection of NiPS_3_@TPP, representative hematoxylin and eosin (H & E) stained images of heart, liver, spleen, lung, and kidney sections. (B) Body weights of the differentially-treated animals over time. (C) Tumor volumes in the different groups and time points (** *P* < 0.01). (D) Therapeutic digital holograms of nude mice. (E) Representative images of tumor tissues.

The therapeutic potential of the NiPS_3_@TPP nano-system was finally tested in a murine tumor-bearing model. As shown by H & E staining (Figure 7A), NiPS_3_@TPP injection did not cause apparent organ damage or inflammation. In addition, almost all blood routine examination (BRT) parameters (Figure S3) and body weight (Figure 7B) of NiPS_3_-treated nude mice were similar to those of the untreated controls. Minimal toxicity of NiPS_3_@TPP at the tested dose indicated that it was a promising biocompatible photosensitizer for *in vivo* biomedical applications. Therefore, we evaluated an enhancement of PDT of the NiPS_3_@TPP targeted system against solid tumors. Perpendicular diameters of the tumors were measured using calipers. Relative tumor size in the different groups and time points were displayed in Figure 7C. When the tumor volumes reached approximately 100 mm^3^, this experiment was conducted within six experimental groups: (1) normal saline, (2) 660 nm laser illumination, (3) NiPS_3_, (4) NiPS_3_ + 660 nm laser, (5) NiPS_3_@TPP, (6) NiPS_3_@TPP + 660 nm laser. After injection of NiPS_3_@TPP, PDT with 660 nm laser light (0.3 W/cm^2^, 10 min) via tail vein was performed 3 times with gaps of 2 days between treatments, as shown in Figure 7D and E, the tumors of the mice treated with the NiPS_3_@TPP nano-system and 660 nm laser irradiation were significantly smaller compared with those in untreated mice. By contrast, NiPS_3_, NiPS_3_@TPP, or 660 nm laser light alone had no significant effect on the tumor volume relative to the control. Therefore, consistent with *in vitro* results, NiPS_3_@TPP can effectively kill tumor cells by PDT using 660 nm laser light stimulation.

## 4 Discussion

PDT is severely limited by the hypoxic environment in a tumor. Current strategies focus primarily on the improvement of the intratumoral O_2_ perfusion, while clinical trials suggest that O_2_ enrichment may promote cancer cell proliferation [3]. The photosensitized ROS generation mechanism of NiPS_3_ should be the photon-generated electron-hole pathway, which can generate O_2_^·−^ and ·OH at the conduction band and valance band, respectively. ·OH generation does not need O_2_, and the O_2_^·−^ can also work in a low O_2_ environment. O_2_^·−^ and ·OH are strong oxidizers that can oxidize and hydroxylate multiple biological macromolecules, including unsaturated fatty acids, sugars, enzymes, or related proteins, which eventually cause extensive cellular damage [51, 52]. The stimulus-response of nanomaterials is promising for precision medicine [53]. As displayed in Figure 2, NiPS_3_ nanosheets absorbed light in the visible and near-infrared range, the absorption wavelength of which can be used as the key to control the biological effect of NiPS_3_ nanosheets. It was non-toxic in absense of being activated by light, while ROS which is highly cytotoxic was generated after light activation. Previous reports suggest that NiPS_3_ nanosheets with only a few layers represent promising catalysts [54]. NiPS_3_ nanosheets generated high levels of ·OH through 660 nm photocatalysis under both normoxic and hypoxic conditions. As shown in Figure 2F, there were 4 distinct characteristic peaks with magnitudes of 1:2:2:1, suggesting the generation of ·OH. When the dissolved O_2_ was removed by N_2_, the generation of ·OH decreased, but were maintained at a steady level. As shown in Figure 2G, the ESR spectrum of NiPS_3_ also demonstrated a separation of electron-hole pairs. Within normoxic conditions, photoexcited electrons from NiPS_3_ mainly captured O_2_ to form O_2_^·−^ and ·OH, while in hypoxic conditions, H+ generated by NiPS_3_ was more likely to oxidize OH− to produce ·OH. The photosensitized ROS generation mechanism of NiPS_3_ should be the photon-generated electron-hole pathway, which can generate O_2_^·−^ and ·OH at the conduction band and valance band, respectively. ·OH generation does not need O_2_, and the O_2_^·−^ can also work in a low O_2_ environments via partial O_2_-recycle in cells. Endocellular ROS generation was further verified using Huh7 cells as a model of cancer (Figure 2I and J). At a particular range of concentrations, compared with the control group, enhanced DCFH-DA green fluorescence was observed, demonstrating intracellular ROS production trigged by 660 nm laser light. The capability of NiPS_3_ to generate ROS even under hypoxia suggests that it represents a highly promising agent for the PDT of solid tumors. It is known that H_2_O_2_ is expressed to a great extent in hypoxic tumor microenvironments [55]. According to previous reports, Ni and Ni complexes can catalyze the reaction to decompose H_2_O_2_ to produce ·OH or O_2_ [56, 57]. We surmise that apart from ·OH, NiPS_3_ catalyzes other reactions such as the decomposition of H_2_O_2_ to produce ROS in a tumor microenvironment.

Effective induced programmed cell death or apoptosis has been a mainstay and goal of clinical cancer therapy. The process of apoptosis can be divided into extrinsic or intrinsic pathways [58]. The extrinsic pathway, dependent on pro-death signals from outside a cell occurs mainly by cell surface receptors, whereas the intrinsic pathway is triggered by mitochondrial events [59, 60]. Mitochondrial targeting in oncotherapy results in a two-pronged cellular damage strategy that destroys the energy supply, stalling proliferation and inducing apoptosis [61]. Nanoparticles with a high net positive charge, such as TPP cations, have the potential to promote endosomal escape and can participate in delivering chemotherapeutic drugs to mitochondria [59]. In the present study, TPP was attached to the surface of NiPS_3_ to target mitochondria. Thus, ROS catalyzed by NiPS_3_ induces mitochondrial outer membrane permeabilization, representing the decisive event that irrevocably commits Huh7 cells to die. In addition, cancer cells have a higher MMP compared with normal cells, conducive to selective targeting [62]. Therefore, we introduced TPP onto the surface of NiPS_3_ to precisely target the mitochondria in Huh7 cells. TPP cations have a strong electric field that promotes their rapid uptake [35]. Since the mitochondrial inner membrane potential is 150–160 mV (negative inside) and that of the cancer cell plasma membrane is 30–60 mV (negative inside), mitochondria can absorb large quantities of cations, and so able to drive the uptake of NiPS_3_@TPP. The Nernst equation indicates that at 37 °C, for every 61.5 mV that the membrane potential increases, the number of singly charged cations increases 10-fold [35, 63]. These properties can promote the absorption of NiPS_3_@TPP nanomaterials by tumor cells. From Figure 4A–C, we demonstrate that the NiPS_3_@TPP nano-system was successfully prepared. The results of scanning electron microscopy (Figure 4D) demonstrated that NiPS_3_@TPP provided superior targeting efficiency than NiPS_3_. Huh7 cells were incubated with NiPS_3_, NiPS_3_@TPP, and NiPS_3_@TPP + 660 nm. In Huh7 cells incubated with NiPS_3_, the nanomaterials were mainly swallowed by lysosomes and randomly dispersed in cytoplasm; the content of nanomaterials in mitochondria was not high. Huh-7 cells had clear and intact nuclear membranes, visible nucleoli, and complete mitochondria without indentations on the inner ridge, all of which indicated healthy, metabolically active cells. In the NiPS_3_@TPP group and the NiPS_3_@TPP + 660 nm group, nanomaterials showed obvious enrichment in mitochondrial, and the nanomaterials were distributed in or around the inner mitochondrial membrane, as indicated by the red triangle in the figure. However, in NiPS_3_@TPP, the mitochondrial inner membrane was intact, while in NiPS_3_@TPP + 660 nm group, the mitochondrial inner membrane collapsed and shrank, with large vacuoles formed in the inner cavity and the outer membrane ruptured, as indicated by the white triangle in the figure. Combine with Figures 2 and 3, we demonstrated that NiPS_3_@TPP was shown to have good biosafety. The results were also verified in animal experiments, as shown in H & E staining (Figure 7A) and routine examination (BRT) parameters (Figure S3). Mitochondria were observed to show apparently morphological and functional destruction such as the loss of cristae, vacuolization, and even mitochondrial membrane rupture after photodynamic therapy following PDT (Figure 4D). Following this, MMP declined and mitochondrial permeability transition pores (MPTP) increased. This process preceded cytochrome-c release from the mitochondria into the cyto-sol, which activated caspase to trigger apoptosis [64]. As shown in Figure 6A–H, the MMP decreased and rate of apoptosis increased after PDT both in normoxic and hypoxic conditions. Bcl-2 is a membrane protein located principally on the outer membrane of mitochondria. Its overexpression prevents cells from undergoing apoptosis, while cytochrome c is required for the initiation of the apoptotic program [65, 66]. Caspase-3 acts as an executioner factor and triggers the apoptosis process by cleaving its protein substrates. Cleavage of caspase-3 is considered a reliable marker of cell death by apoptosis [67, 68]. As shown by the results of Western blotting (Figure 6J), cytochrome c and cleaved caspase-3 were the highest in the NiPS_3_@TPP + 660 nm laser group, but Bcl-2 expression was the least among these three treatments. DNA damaging agents, such as ROS may activate both membrane death receptors and endogenous mitochondrial damage pathways leading to apoptosis [69]. As shown in Figure 6I, compared to the control group, the NiPS_3_@TPP + 660 nm group clearly exhibited double-strand breaks (DSBs), indicating severe photodynamic damage.

Following *in vivo* therapy for specific durations, as shown in Figure 7A, the heart, liver, spleen, lungs, and kidneys in the experimental group displayed no pathological changes compared with the control group. As shown in Figure 7B, there was a slight increase in body weight, and furthermore, no animals died in any *in vivo* experiment, suggesting that there were negligible toxic side effects for these treatments. This conclusion is consistent with previous *in vitro* experiments. As shown in Figure 7C–E, the tumor volumes of the photodynamic groups (Groups 4 and 6) were significantly smaller than those in other groups. The therapeutic effect of the targeted modification group (Group 6) was significantly superior to that of the non-targeted modification group (Group 4). In view of the observations described above, we conclude that the NiPS_3_@TPP has potential for applications in nano-medicine.

## 5 Conclusions

We developed a photosensitizer NiPS_3_@TPP that can produce ROS independent of oxygen and thus can kill cancer cells. The photosensitized ROS generation mechanism of NiPS_3_ is the photon-generated electron-hole pathway, which can generate O_2_^·−^ and ·OH at the conduction band and valance band, respectively. More crucial is that ·OH generation doesn’t need O_2_, and the O_2_^·−^ can also work in a low O_2_ environment, and depleting oxygen in tumor cells. In a hepatoma tumor model, NiPS_3_@TPP system-based PDT significantly inhibited tumor growth and displayed low biotoxicity. Thus, NiPS_3_ is a promising next-generation photosensitizer representing a simple and effective nano-enhancer for PDT.

## Supplementary Material

Supplementary Material Details
