# Supplementary material for: Novel photodynamic therapy using two-dimensional NiPS3 nanosheets that target hypoxic microenvironments for precise cancer treatment
Source: Nanophotonics. 2022 Dec 1;12(1):81–98. doi: 10.1515/nanoph-2022-0520 (PMC11501689; doi:10.1515/nanoph-2022-0520)
Supplement: Supplementary file 1 — Supplementary Material Details [file j_nanoph-2022-0520_suppl.docx]

**Supplementary Information**

**
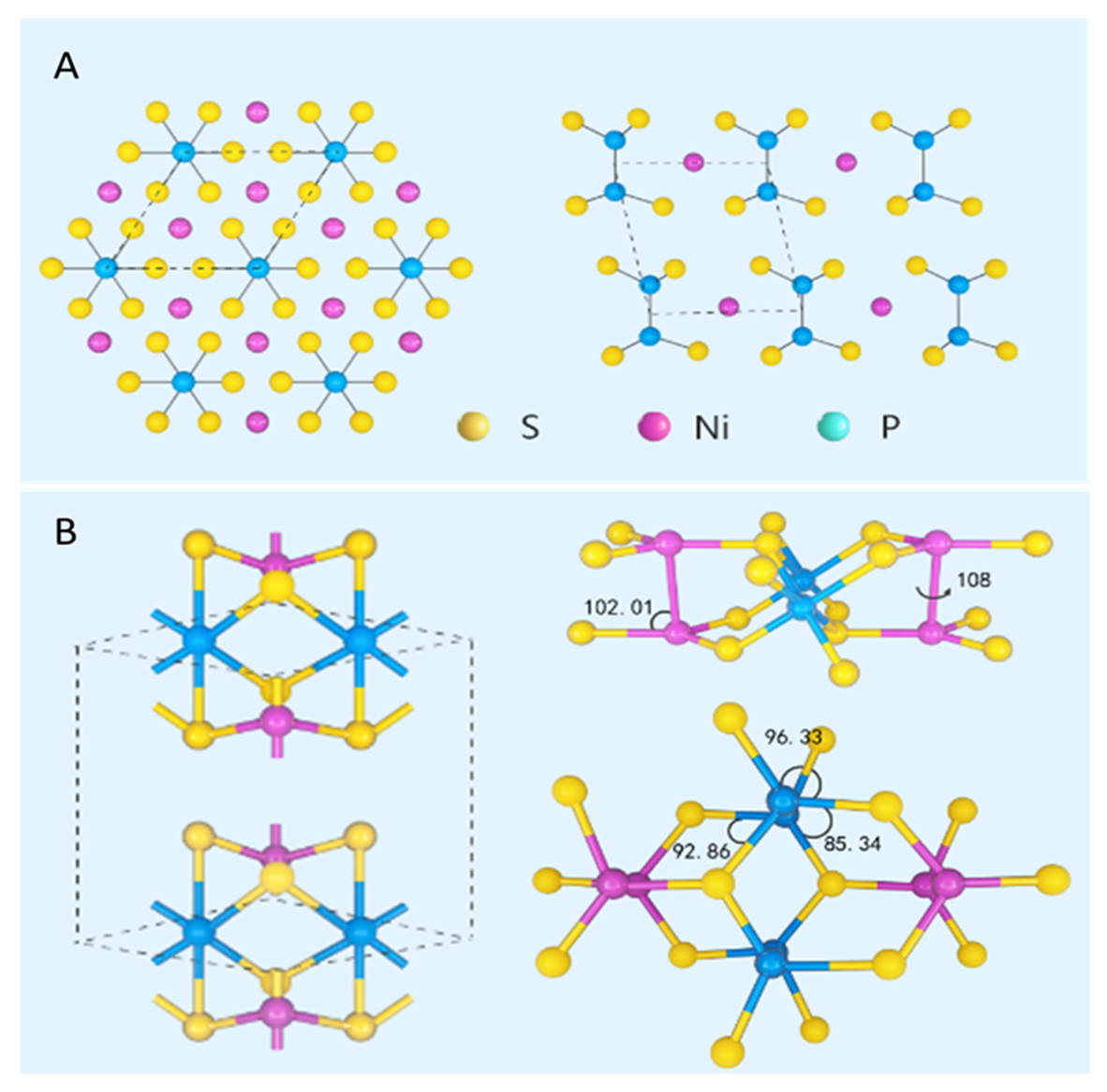
**

**Supplementary Figure 1.** (A) Top and side view of the crystal structure of monolayer NiPS_3_. The green, red and yellow spheres represent the Ni, P and S atoms, respectively. (B) Crystal structure of NiPS_3_.

| Phase date | |
| --- | --- |
| Formula sum | NiPS_3_ |
| Formula Weight | 185.87 |
| Crystal system | triclinic |
| Space-group | P1(1) |
| Cell parameters | a = 5.812 Å, b = 10.070 Å, c = 6.632 Å, α=90.00 Å，β=106.98 Å，γ=90.00^。^ |
| Space group | C2/m |
| Cell ratio | a/b=0.577，b/c=1.518，c/a=1.141 |
| Cell volume | 371.2 Å^3^ |
| Z | 2 |
| Density(gm/cc) | 3.300 |
| Formation energy(eV | -0.601 |

**Supplementary Table 1.** In the structure model of NiPS_3_, Ni ions immobilize [P_2_S_6_]^4^- framework and the metal layers sandwiched by distorted octahedral S layers, forming 2D architectures that are stacked ogether via van der Waals force. A regular pattern of octahedrally coordinated sites is formed. 2/3 of the octahedral centers are filled by Ni^2+^ cations. The remaining 1/3 are filled by P-P imers which covalently bond to the sulfur atoms with each P atom having tetrahedral oordination with 3 S atoms and 1 P atom, forming an ethane-like [P_2_S_6_]^4^- unit. Each S atoms bonded to a single P atom and coordinated by two Ni sites.

| Item | Compare | Reference |
| --- | --- | --- |
| intermediate range of bandgaps | ≈1.6 Ev | [1](#_ENREF_1) |
| in-plane stiffness C2D (N/m)，stability | FePS_3_＞MnPS_3_ NiPS_3_＞ZnPS_3_＞CdPS_3_ | [2](#_ENREF_2) |
| evolution reaction activity by MPX3 based photocatalysts | NiPS_3_> FePS_3_> CdPS_3_ > MnPS_3_> ZnPS_3_ | [3](#_ENREF_3) |
| P-P bond length | NiPS_3_>FePS_3_>ZnPS_3_ >MnPS_3_>CdPS_3_ | [3](#_ENREF_3) |
| M-S bond length | NiPS_3_>FePS_3_>ZnPS_3_ >MnPS_3_>CdPS_3_ | [3](#_ENREF_3) |
| Cytotoxicity of layered metal phosphorus chalcogenides (MPXY) nanoflakes | CoPS_3_＞FePS_3_＞NiPS_3_ ≈Black phosphorus | [4](#_ENREF_4) |

**Supplementary Table 2.** **Related parameters of NiPS_3_**

**
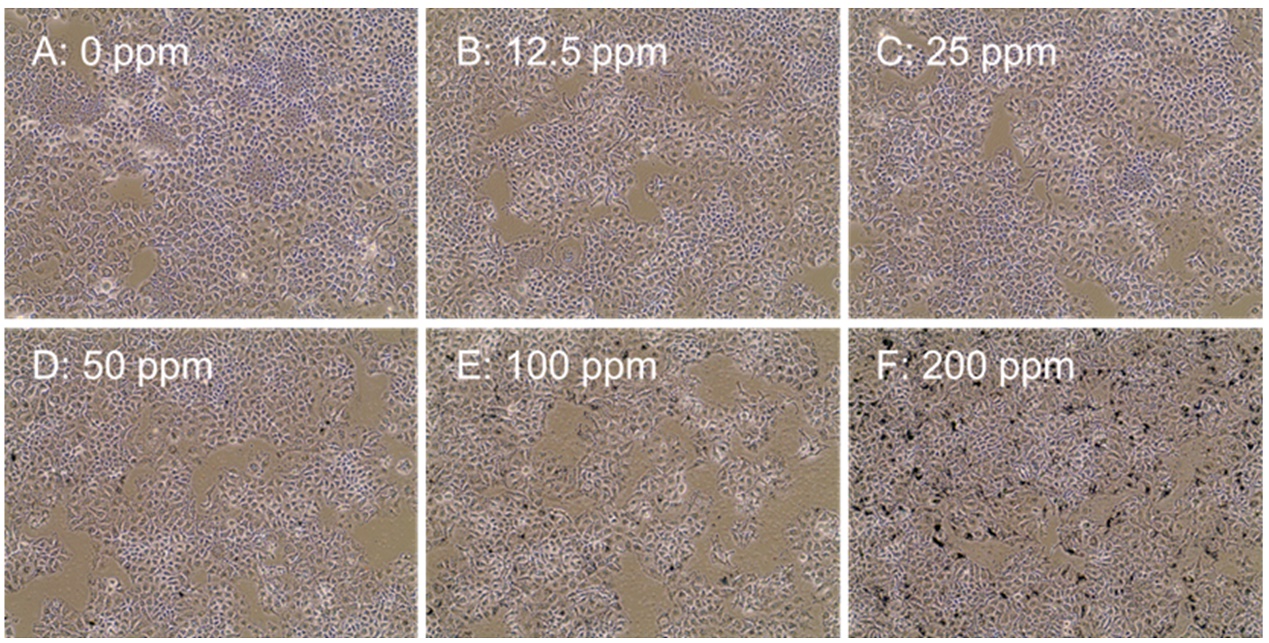
**

**Supplementary Figure 2.** The effects of different concentrations of NiPS3@TPP on cell growth were observed under an inverted microscope. It can be seen that there is no obvious toxic for NiPS_3_ nanosheet to the cell.

**
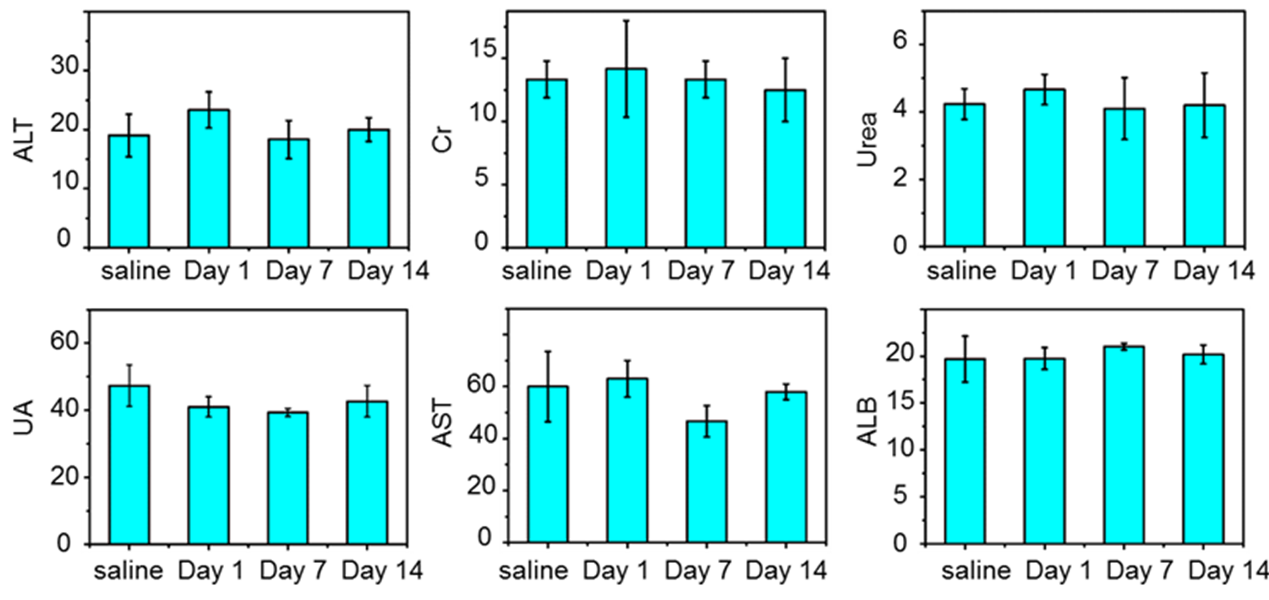
**

**Supplementary Figure 3:** Blood biochemistry analyses. The mice were intravenously injected with PBS or NiPS_3_-TPP or (dosage: 3 mg NiPS_3_ equiv./kg). 0.8 mL Blood was sampled from rat eyes at specific time, and immediately centrifuged at 5000 rpm for 3 mins to obtain blood serum for blood biochemistry analysis. Blood levels of creatinine(Cr), uric acid(UA), alanine aminotransferase (ALT), aspartate aminotransferase (AST) and albumin(ALB) were examined, using saline treated healthy mice as a control (n=3).

**References**

1. Ma Z, Wang F, Dou M, Yao Q, Wu F, Kan E. Boosting the high-capacity with multi-active centers: A first-principles investigation of NiPS3 monolayer as an anode material. Applied Surface Science. 2019;**495**:143534.

2. Mayorga-Martinez CC, Sofer Z, Sedmidubsky D, Huber S, Eng AY, Pumera M. Layered Metal Thiophosphite Materials: Magnetic, Electrochemical, and Electronic Properties. ACS applied materials & interfaces. 2017;**9**(14):12563-73.

3. Barua M AMM, Vishnoi P , et al. . Photochemical HER activity of layered metal phospho-sulfides and –selenides. Journal of Materials Chemistry A. 2019;**7**. DOI:10.1039/C9TA06044H

4. Latiff NM, Mayorga-Martinez CC, Khezri B, Szokolova K, Sofer Z, Fisher AC, et al. Cytotoxicity of layered metal phosphorus chalcogenides (MPXY) nanoflakes; FePS3, CoPS3, NiPS3. FlatChem. 2018;**12**:1-9.
